# Evaluation of early liquid drinking after radical gastrectomy in gastric cancer: a Chinese multicenter propensity score matching analysis

**DOI:** 10.1093/gastro/goad029

**Published:** 2023-06-08

**Authors:** Yue Zhang, Kaixiong Tao, Jinlong Yu, Chao Chen, Quan Zheng, Sanlin Lei, Xiaogang Zhong, Lixin Liu, Wei Wang, Qiang Wang, En Li, Yuwen Luo, Guanrong Zhang, Xingyu Feng, Yong Li, Junjiang Wang

**Affiliations:** Department of Gastrointestinal Surgery, Department of General Surgery, Guangdong Provincial People’s Hospital, Guangdong Academy of Medical Sciences, Southern Medical University, Guangzhou, Guangdong, P. R. China; Shantou University Medical College, Shantou, Guangdong, P. R. China; Department of Gastrointestinal Surgery, Union Hospital, Tongji Medical College, Huazhong University of Science and Technology, Wuhan, Hubei, P. R. China; Department of General Surgery, Zhujiang Hospital of Southern Medical University, Guangzhou, Guangdong, P. R. China; Department of Gastrointestinal Surgery, Huizhou Municipal Central Hospital, Huizhou, Guangdong, P. R. China; Department of Gastrointestinal Surgery, Guangdong Second Provincial General Hospital, Guangzhou, Guangdong, P. R. China; Department of Gastrointestinal Surgery, The Second Xiangya Hospital of Central South University, Changsha, Hunan, P. R. China; Department of Surgical Treatment of Gastrointestinal Hernia and Fistula, The People’s Hospital of Guangxi Zhuang Autonomous Region, Guangxi Academy of Medical Sciences, Nanning, Guangxi, P. R. China; Department of General Surgery, The Third Affiliated Hospital of Southern Medical University, Guangzhou, Guangdong, P. R. China; Department of Gastrointestinal Surgery, The Second Affiliated Hospital of Guangzhou University of Chinese Medicine, Guangdong Provincial Hospital of Traditional Chinese Medicine, Guangzhou, Guangdong, P. R. China; Department of Gastrointestinal Surgery, Guangzhou First People’s Hospital, Guangzhou, Guangdong, P. R. China; Department of Gastrointestinal Surgery, Meizhou People’s Hospital, Meizhou, Guangdong, P. R. China; Department of General Surgery, Zhujiang Hospital of Southern Medical University, Guangzhou, Guangdong, P. R. China; lnformation and Statistics Center, Guangdong Provincial People’s Hospital, Guangdong Academy of Medical Sciences, Southern Medical University, Guangzhou, Guangdong, P. R. China; Department of Gastrointestinal Surgery, Department of General Surgery, Guangdong Provincial People’s Hospital, Guangdong Academy of Medical Sciences, Southern Medical University, Guangzhou, Guangdong, P. R. China; Shantou University Medical College, Shantou, Guangdong, P. R. China; Department of Gastrointestinal Surgery, Department of General Surgery, Guangdong Provincial People’s Hospital, Guangdong Academy of Medical Sciences, Southern Medical University, Guangzhou, Guangdong, P. R. China; Shantou University Medical College, Shantou, Guangdong, P. R. China; Department of Gastrointestinal Surgery, Department of General Surgery, Guangdong Provincial People’s Hospital, Guangdong Academy of Medical Sciences, Southern Medical University, Guangzhou, Guangdong, P. R. China; Shantou University Medical College, Shantou, Guangdong, P. R. China

**Keywords:** gastric cancer, early liquid drinking, traditional liquid drinking, gastrointestinal function, propensity score matching

## Abstract

**Background:**

Enhanced recovery after surgery is used in gastrointestinal surgery. This study aimed to access the effects of early liquid drinking (ELD) on gastrointestinal function recovery in patients with gastric cancer (GC) who underwent radical gastrectomy, as high-quality evidence on the outcomes of ELD after gastrectomy is currently lacking.

**Methods:**

Clinicopathological data of patients with GC from 11 centers were retrospectively analysed. Clinical outcomes were investigated in 555 patients, including 225 who started drinking liquid within 48 h (ELD group) of surgery and 330 who started drinking liquid after flatus resumption (traditional liquid drinking [TLD] group). Propensity score matching (PSM) analysis was performed using a match ratio of 1:1 and 201 patients were selected from each group for the analysis. Primary outcome was time to first passage of flatus. Secondary outcomes included time to first defecation, post-operative hospitalization days, occurrence of short-term post-operative complications, and hospitalization costs.

**Results:**

After PSM, baseline characteristics were not significantly different between the two groups. The time to first flatus (2.72 ± 1.08 vs 3.36 ± 1.39 days), first defecation (4.34 ± 1.85 vs 4.77 ± 1.61 days), and post-operative hospital stay (8.27 ± 4.02 vs 12.94 ± 4.43 days) were shorter in the ELD group than in the TLD group (all *P *<* *0.05). The ELD group had lower hospitalization costs than the TLD group ([7.83 ± 2.44 vs 8.78 ± 3.41] × 10^4^ RMB, *P *=* *0.041). No significant differences were observed in the incidence of post-operative complications.

**Conclusions:**

Compared with TLD, post-operative ELD could promote rapid recovery of gastrointestinal function and reduce hospitalization costs; moreover, ELD does not increase the risk of post-operative complications.

## Introduction

Gastric cancer (GC) is one of the most common malignancies worldwide, with a particularly high incidence in East Asia [1]. Recently, it has been the second leading cause of cancer-related mortality in China [2]. Surgery remains the mainstay of treatment for advanced GC [3]. Recent studies have shown that early liquid drinking (ELD) is safe and feasible for patients who undergo gastrectomy and can improve their nutritional status [4–6]. The Enhanced Recovery After Surgery (ERAS) protocol is an evidence-based multidisciplinary perioperative approach aimed at reducing hospital stays, decreasing the incidence of surgery-related complications, maintaining post-operative physiological function, and promoting early recovery. The ERAS protocol includes a number of preoperative, intraoperative, and post-operative optimization measures, including ELD.

In the past few decades, avoiding ELD after gastrointestinal surgery has been a common practice to avoid complications, such as post-operative intestinal obstruction; however, it is now considered unnecessary and could even be harmful in certain conditions. In a recent meta-analysis comprising six randomized–controlled studies, ELD within 24 h after surgery was found to reduce the mortality rate and hospital stay without increasing the risk of anastomotic complications [7]. Radical gastrectomy is one of the most complex procedures in the field of gastrointestinal surgery. However, early oral feeding after radical gastrectomy has not been widely accepted by GC patients, or even some gastrointestinal surgeons [8], mainly due to safety and risk-to-benefit considerations, of which the occurrence of anastomotic complications is of most concern. According to a randomized–controlled trial (RCT) from Japan, ELD was shown to be associated with a potential benefit for total/proximal gastrectomy (TG/PG) patients, but due to the relatively small sample size of that study, further verification is awaited [9]. Although some related studies on ELD have been conducted in China [10–12], most of them were single-center retrospective observational studies and lacked high-level clinical research evidence.

This study assessed the effects of ELD and traditional liquid drinking (TLD) on the recovery of gastrointestinal function in patients with GC who underwent radical gastrectomy at 11 medical centers, with the hope of providing accurate references to oncologists and patients to improve the perioperative measures of GC.

## Materials and methods

### Study settings

This multicenter retrospective study was based on a collaborative effort of 11 centers. Clinicopathological data from GC patients who underwent radical gastrectomy at these institutions between January 2015 and December 2017 were collected and assessed. This retrospective study was approved by the Institutional Review Board of all participating centers, and it adhered to the Declaration of Helsinki (No. GDREC2020143H[R2]). The need for informed consent from all patients was waived owing to the retrospective nature of the study.

### Study population

**Figure 1.**
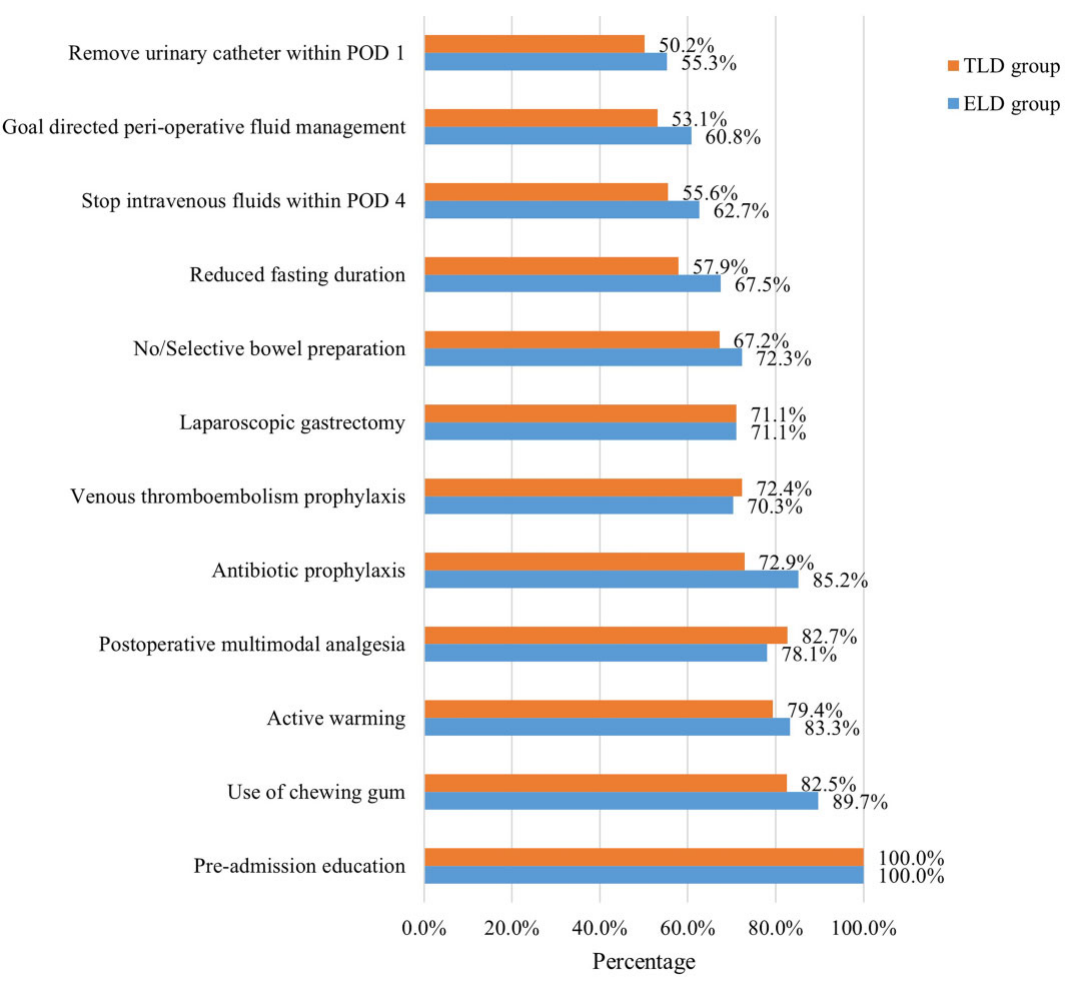
Compliance with ERAS protocol elements. ERAS, enhanced recovery after surgery; ELD, early liquid drinking; TLD, traditional liquid drinking; POD, post-operative day.

**Figure 2.**
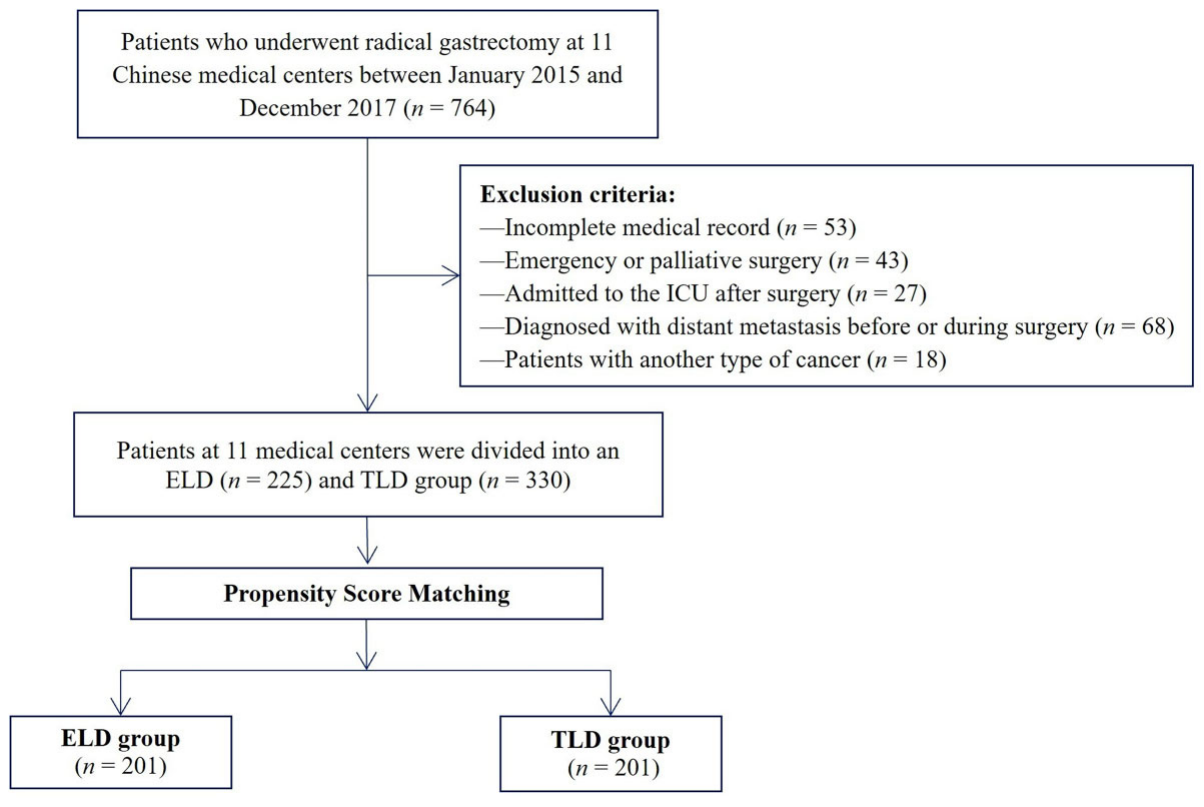
Flowchart of patient inclusion in this study. ICU, intensive care unit; ELD, early liquid drinking; TLD, traditional liquid drinking.

Inclusion criteria were as follows: (i) pathologically confirmed gastric adenocarcinoma; (ii) patients who underwent radical resection; (iii) patients who had physical status classification system graded from I to III according to the American Society of Anesthesiologists (ASA); and (iv) all patients and their families who agreed to receive the perioperative ERAS protocol in Figure 1. Exclusion criteria were as follows: (i) patients who underwent emergency or palliative surgeries; (ii) patients suffering from serious infectious diseases, such as tuberculosis and/or cardiopulmonary infarction, and other serious uncontrolled organic diseases; (iii) patients diagnosed with distant metastasis before or during surgery; (iv) simultaneous occurrence of other types of cancer; (v) patients admitted into the intensive care unit after surgery; and (vi) incomplete medical records. A flowchart of the patient inclusion process is shown in Figure 2.

### Data collection

As with all clinical research, our study group brought together clinicians and statisticians to work together to design the best study protocol that will assess and compare the efficacy of ELD and TLD. Patients’ basic clinical information, such as gender, age, body mass index (BMI), ASA physical status classification, nutritional status, preoperative mechanical bowel preparation, tumor location, tumor differentiation, and post-operative pathological tumor staging, were collected. Pathological tumor stage was according to the third-edition National Comprehensive Cancer Network (NCCN) GC staging system [13]. Surgical and post-operative recovery information including surgical approaches (laparoscopic surgery, open surgery, or laparoscopy converted to open surgery), extent of resection (distal, proximal, or total gastrectomy), operation duration, and blood loss were also collected.

The primary outcome of this study was the time to first passage of flatus, which reflects post-operative recovery of gastrointestinal function. Secondary outcomes included time to first defecation, post-operative hospitalization days, hospitalization cost, and occurrence of short-term post-operative complications (defined as complications occurring within 30 days after surgery; Clavien–Dindo classification ≥ III was considered a significant complication [14], i.e. pulmonary infection, incision infection, post-operative gastroparesis, anastomotic leakage, bowel obstruction, abdominal infection, abdominal bleeding, etc.).

### Perioperative assessment and patient grouping

A preoperative diagnosis was made through endoscopy, biopsy, and enhanced computed tomography (CT). All the patients underwent standardized preoperative assessment including abdominal CT, chest radiography, blood routine, tumor marker assessment (i.e., carcinoembryonic antigen [CEA] and carbohydrate antigen 19–9 [CA19-9]), liver and kidney function tests, endocrinological evaluation, etc.

The cases who started oral feeding within 48 h of gastrectomy were classified into an ELD group and those who started oral feeding after the resumption of flatus were classified into a TLD group. We suggested that post-operative patients should start oral feeding according to the protocol that is shown in Table 1 as soon as possible. However, because of the higher incidence of post-operative complications after TG/PG, the feeding strategy can be adjusted according to blood inspection, drainage characteristics, esophagogram results, and patient tolerance to promote gastrointestinal function recovery under the premise of safety. All the patients received similar counseling, including preoperative education and nutritional evaluation during the perioperative period. They were advised to avoid drinking liquid for 2 h and to fast for 6 h before their surgery, and were administered prophylactic antibiotics intravenously 30 min before skin incision on the operation day. Surgical treatment included standard gastrectomy with D2 lymph node dissection, according to the guidelines of the Japanese Gastric Cancer Association [15]. Multimodal measures for pain management were used and the urinary catheter was removed on post-operative Day 1. Indwelling nasogastric tubes were not routinely used after surgery [16], reducing the number of abdominal drainage tubes and minimizing the duration of their placement.

**Table 1.** Dietary protocol of the early liquid drinking group

Time point	Protocol
Day of surgery	Attempt to drink warm water (<50 mL/h) 6 h after surgery was encouraged
Post-operative Day 1 or 2	Total oral fluid intake increased up to 200 mL per day (<50 mL/h), water was given
Post-operative Day 2 or 3	Total oral fluid intake increased up to 500 mL (<50 mL/h), liquid diet (such as small amounts of rice soup) was started
Post-operative Day 3 or 4	Total oral fluid intake increased up to 1,000 mL (<100 mL/h), intravenous fluid volume was gradually reduced
Post-operative Day 4 or 5	Small amounts of semi-liquid foods (such as porridge, noodles, or other soft foods), intravenous fluids stopped if possible
Post-operative Day 5 or 6	Frequent small amounts of oral fluids with gradual transition to total semi-liquid diet and soft foods

All the patients were divided into two groups based on their surgical treatment, namely the distal gastrectomy (DG) group and the TG/PG group [17]. The operative and post-operative outcomes were retrospectively analysed. Discharge criteria are as follows: (i) results of the auxiliary examination indicated that no complications or post-operative complications were effectively controlled; (ii) solid or semi-solid foods were tolerable and oral feeding provided sufficient energy requirements; (iii) no fluid therapy; (iv) gastrointestinal function was restored; (v) abdominal drainage tube was removed; and (vi) the patient agreed to be discharged.

### Statistical analysis

The propensity score matching (PSM) method was employed to reduce the possibility of selection bias in retrospective observational studies and to adjust for significant differences in the baseline characteristics of the enrolled patients (gender, age, BMI, ASA grade, tumor location, surgical approaches, and extent of resection). A logistic regression model was used to calculate the propensity score for each individual patient. The matching algorithm was the nearest neighbor, with a match ratio of 1:1 and caliper value of 0.05. After the PSM, 201 patients were included in each treatment group.

Statistical analyses were performed using SPSS software (version 25.0). Quantitative data with normal distribution and homogeneous variances are presented as mean ± standard deviation and the differences between the two groups were determined using the independent sample *t*-test. Non-normal distribution data are described as the median (quartile) and the Wilcoxon rank-sum test was used to compare differences between the groups. The counting data were described by frequency and percentage, and the chi-square test or Fisher’s exact probability method was used to compare frequencies between the groups. Statistical significance was set at *P *<* *0.05.

## Results

### Baseline characteristics

**Figure 3.**
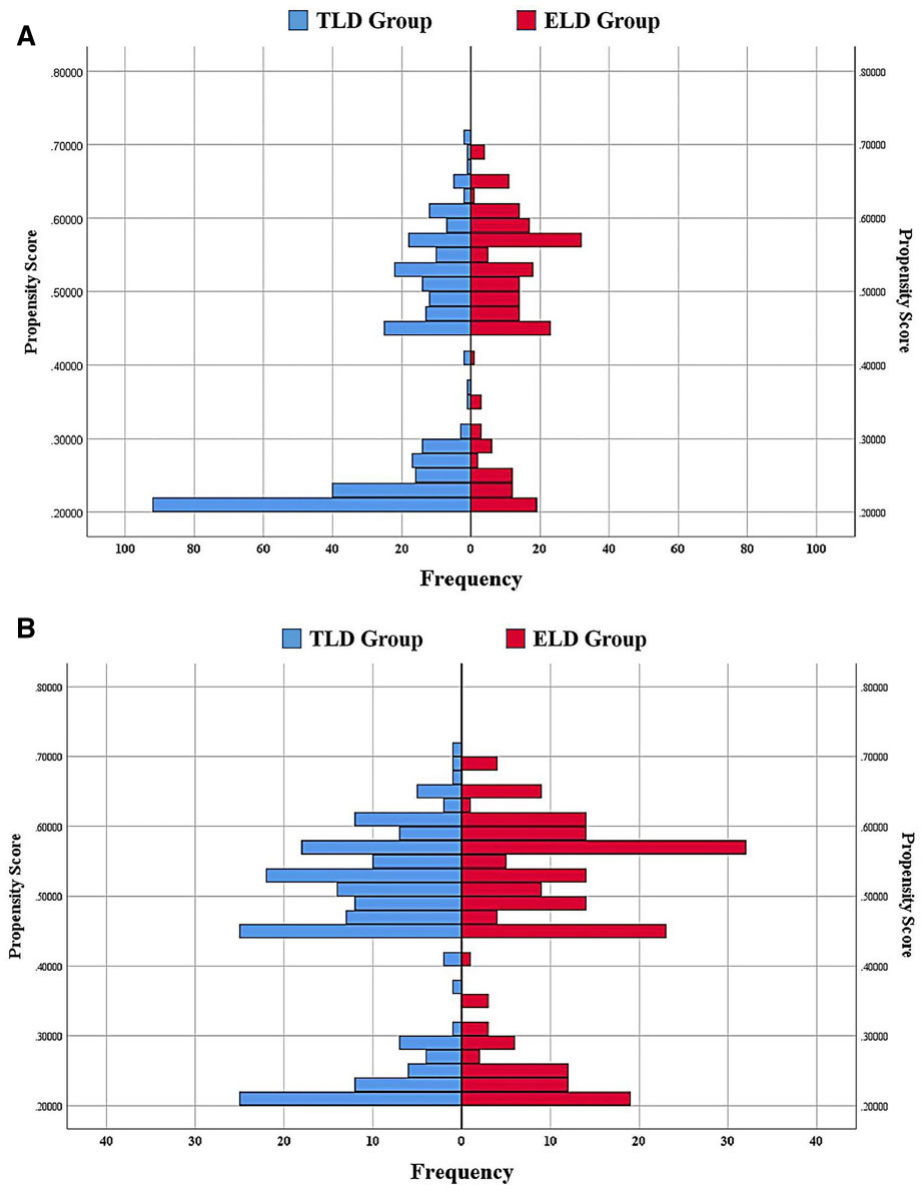
The distribution of propensity scores before and after matching. (A) Before PSM. (B) After PSM. ELD, early liquid drinking; TLD, traditional liquid drinking; PSM, propensity score matching.

A total of 555 patients were eligible for this study, of whom 225 (40.5%) were classified into the ELD group and 330 (59.5%) into the TLD group. Baseline characteristics are shown in Table 2. Before the PSM, there were significant differences in gender (*P *=* *0.042), preoperative mechanical bowel preparation (*P *<* *0.001), surgical approach (*P *<* *0.001), and lymph node stage (N stage) (*P *=* *0.032) between the two groups. However, there were no statistically significant differences in age, BMI, nutritional assessment, ASA grade, tumor location, extent of resection, tumor differentiation, or tumor stage (T stage) between the two groups (all *P *>* *0.05). After the PSM, 201 patients were selected from each group and the baseline characteristics were well balanced between the two matched groups (Table 2). The distribution of propensity scores before and after matching is shown in Figure 3. Perioperative ERAS interventions are shown in Table 3.

**Table 2.** Baseline clinicopathological characteristics of the ELD and TLD groups before and after propensity score matching

Characteristic	Before matching		*P_1_*-value	Standard difference before matching	After matching		*P_2_*-value	Standard difference after matching
	ELD group (*n* = 225)	TLD group (*n* = 330)			ELD group (*n* = 201)	TLD group (*n* = 201)		
Gender, *n* (%)			0.042^a^	0.176			0.148^a^	0.155
Male	141 (62.7)	234 (70.9)			119 (59.2)	134 (66.7)		
Female	84 (37.3)	96 (29.1)			82 (40.8)	67 (33.3)		
Age, *n* (%)			0.448^a^	0.073			0.135^a^	0.160
<60 years	107 (47.6)	169 (51.2)			94 (46.8)	110 (54.7)		
≥60 years	118 (52.4)	161 (48.8)			107 (53.2)	91 (45.3)		
BMI, *n* (%)			0.441^a^	0.074			0.303^a^	0.113
<23 kg/m^2^	133 (59.1)	207 (62.7)			120 (59.7)	131 (65.2)		
≥23 kg/m^2^	92 (40.9)	123 (37.3)			81 (40.3)	70 (34.8)		
Presence of malnutrition, *n* (%)	27 (12.0)	38 (11.5)	0.968^a^	0.015	24 (11.9)	26 (12.9)	0.880^a^	0.030
Mechanical bowel preparation, *n* (%)	56 (24.9)	41 (12.4)	< 0.001^a^	0.324	51 (25.4)	38 (18.9)	0.149^a^	0.156
Surgical approaches, *n* (%)			< 0.001^a^	0.654			1.000^a^	< 0.001
Laparoscopy	167 (74.2)	144 (43.6)			143 (71.1)	143 (71.1)		
Open	58 (25.8)	186 (56.4)			58 (28.9)	58 (28.9)		
Tumor location, *n* (%)			0.711^a^	0.072			0.098^a^	0.216
Proximal	54 (24.0)	77 (23.3)			50 (24.9)	34 (16.9)		
Middle	57 (25.3)	94 (28.5)			48 (23.9)	61 (30.3)		
Distal	114 (50.7)	159 (48.2)			103 (51.2)	106 (52.8)		
Gastrectomy type, *n* (%)			0.226^a^	0.150			0.683^a^	0.087
Proximal	12 (5.3)	25 (7.6)			11 (5.5)	8 (4.0)		
Distal	135 (60.0)	175 (53.0)			121 (60.2)	118 (58.7)		
Total	78 (34.7)	130 (39.4)			69 (34.3)	75 (37.3)		
ASA status, *n* (%)			0.151^a^	0.170			0.063^a^	0.233
I	71 (31.6)	84 (25.4)			67 (33.3)	67 (33.3)		
II	143 (63.5)	220 (66.7)			125 (62.2)	113 (56.2)		
III	11 (4.9)	26 (7.9)			9 (4.5)	21 (10.5)		
Tumor differentiation, *n* (%)			0.811^a^	0.086			0.333^a^	0.185
Undifferentiated	1 (0.4)	3 (0.9)			0 (0.0)	3 (1.5)		
Poor	132 (58.7)	203 (61.5)			114 (56.7)	118 (58.7)		
Moderate	80 (35.6)	108 (32.7)			76 (37.8)	69 (34.3)		
High	12 (5.3)	16 (4.9)			11 (5.5)	11 (5.5)		
pT stage, *n* (%)			0.792^a^	0.113			0.753^a^	0.138
pT1	41 (18.2)	55 (16.7)			41 (20.4)	36 (17.9)		
pT2	34 (15.1)	42 (12.7)			29 (14.4)	24 (11.9)		
pT3	58 (25.8)	81 (24.5)			52 (25.9)	55 (27.4)		
pT4a	72 (32.0)	117 (35.5)			60 (29.9)	70 (34.8)		
pT4b	20 (8.9)	35 (10.6)			19 (9.4)	16 (8.0)		
pN stage, *n* (%)			0.032^a^	0.287			0.188^a^	0.249
pN0	85 (37.8)	113 (34.2)			83 (41.3)	77 (38.3)		
pN1	41 (18.2)	98 (29.7)			28 (13.9)	46 (22.9)		
pN2	41 (18.2)	55 (16.7)			41 (20.4)	34 (16.9)		
pN3a	41 (18.2)	42 (12.7)			36 (17.9)	29 (14.4)		
pN3b	17 (7.6)	22 (6.7)			13 (6.5)	15 (7.5)		
Operating time, min, mean ± SD	285.5 ± 82.0	267.3 ± 79.1	0.009^b^		282.9 ± 83.1	277.5 ± 83.9	0.515^b^	
Intraoperative bleeding, mL, mean (range)	150 (20–800)	200 (20–1,300)	<0.001^b^		150 (20–800)	150 (20–1,300)	0.231^b^	

BMI, body mass index; ASA, American Society of Anesthesiologists; ELD, early liquid drinking; TLD, traditional liquid drinking; SD, standard deviation.

a Chi-square test value.

b *t*-test value.

**Table 3.** Perioperative Enhanced Recovery After Surgery (ERAS) interventions

Preoperative	Intraoperative	Post-operative
Pre-admission education^a^	Active warming	Early oral nutrition^a^
Antibiotic prophylaxis	Opioid-sparing technique	Early ambulation
Reduced fasting duration	Surgical techniques^a^	Early catheter removal
Carbohydrate loading	Avoidance of prophylactic nasogastric tubes	Use of chewing gum
No/selective bowel preparation	Pain and nausea management^a^	–
Venous thromboembolism prophylaxis	Goal-directed perioperative fluid management^a^	
–	Reduce the number and duration of drainage tubes	

a Core items in ERAS management.

### Post-operative outcomes after PSM

Post-operative outcomes of patients in the two groups after the PSM are shown in Tables 4 and 5. The time to first flatus, defecation, and post-operative hospital stay were significantly shorter in the ELD group than those in the TLD group (all *P *<* *0.05). Hospitalization costs were also lower in the ELD group (*P *<* *0.05). There was no significant difference in the incidence of post-operative complications between the groups (*P *>* *0.05).

**Table 4.** Post-operative outcomes of the whole groups after propensity score matching

Outcome	ELD group (*n* = 201)	TLD group (*n* = 201)	*P*-value
First flatus, days, mean ± SD	2.72 ± 1.08	3.36 ± 1.39	<0.001^b^
First defecation, days, mean ± SD	4.34 ± 1.85	4.77 ± 1.61	0.014^b^
Post-operative hospital stay, days, mean ± SD	8.27 ± 4.02	12.94 ± 4.43	<0.001^b^
Hospitalization costs, × 10^4^ RMB, mean ± SD	7.83 ± 2.44	8.78 ± 3.41	0.041^b^
Presence of significant complications, *n* (%)	13 (6.5)	18 (9.0)	0.455^a^
Unplanned 30-day readmissions, *n* (%)	1 (0.5)	1 (0.5)	1.000^a^

ELD, early liquid drinking; TLD, traditional liquid drinking; SD, standard deviation.

a Chi-square test value.

b *t*-test value.

**Table 5.** Comparison of post-operative complications between ELD group and TLD group after propensity score matching.

Complication	Total (*n* = 402)	ELD group (*n* = 201)	TLD group (*n* = 201)
All significant complications^a^	31 (7.7)	13 (6.5)	18 (9.0)
Abdominal infection	8 (2.0)	3 (1.5)	5 (2.5)
Pulmonary infection	6 (1.5)	2 (1.0)	4 (2.0)
Anastomotic leakage	6 (1.5)	3 (1.5)	3 (1.5)
Abdominal bleeding	5 (1.2)	3 (1.5)	2 (1.0)
Bowel obstruction	4 (1.0)	1 (0.5)	3 (1.5)
Incision infection	2 (0.5)	1 (0.5)	1 (0.5)

ELD, early liquid drinking; TLD, traditional liquid drinking.

a Clavien–Dindo classification ≥ III was considered a significant complication.

### Operative and post-operative subgroup analysis based on the extent of resection

In the subgroup analysis of patients who underwent DG or TG/PG, there were no statistically significant differences in gender, age, BMI, ASA grade, nutritional assessment, preoperative mechanical bowel preparation, tumor differentiation, and post-operative pathological staging between the two groups (all *P *>* *0.05). For patients who underwent DG, the time to first defecation tended to be shorter in the ELD group than in the TLD group, but the difference was not statistically significant (*P *>* *0.05). Furthermore, irrespective of the type of gastrectomy performed, post-operative ELD was associated with a significant shortening of the time to flatus and the post-operative hospital stay (*P *<* *0.05), and there was no increase in post-operative complications (Table 6).

**Table 6.** The operative and post-operative outcomes of the subgroup analysis

Outcome	DG group		*P*-value	TG/PG group		*P*-value
	ELD group (*n* = 121)	TLD group (*n* = 118)		ELD group (*n* = 80)	TLD group (*n* = 83)	
Operating time, min, mean ± SD	258.7 ± 63.2	252.9 ± 62.9	0.479^b^	319.5 ± 95.6	312.4 ± 97.1	0.637^b^
Intraoperative bleeding, mL, mean (range)	100 (20–1,000)	150 (20–400)	0.279^b^	200 (20–800)	200 (50–1,300)	0.531^b^
First flatus, days, mean ± SD	2.74 ± 1.00	3.19 ± 1.39	0.004^b^	2.69 ± 1.20	3.59 ± 1.36	<0.001^b^
First defecation, days, mean ± SD	4.40 ± 1.77	4.55 ± 1.45	0.486^b^	4.25 ± 1.98	5.08 ± 1.76	0.005^b^
Post-operative hospital stay, days, mean ± SD	7.88 ± 3.57	12.47 ± 4.31	<0.001^b^	8.86 ± 4.58	13.60 ± 4.55	<0.001^b^
Presence of significant complications, *n* (%)	5 (4.13)	8 (6.78)	0.537^a^	8 (10.00)	10 (12.05)	0.867^a^
Unplanned 30-day readmissions, *n* (%)	1 (0.83)	–	–	–	1 (1.20)	–

ELD, early liquid drinking; TLD, traditional liquid drinking; DG, distal gastrectomy; TG, total gastrectomy; PG, proximal gastrectomy; SD, standard deviation.

a Chi-square test value.

b *t*-test value.

## Discussion

As one of the most common malignant tumors worldwide, GC seriously affects public health [1, 18]. With improvements in the surgical treatment of GC management, the survival rate of patients has gradually improved. Currently, improving the quality of life of patients is the new focus and primary goal of researchers while still striving for new ways to improve cancer patients' treatments and outcomes. For patients with GC, diet is an important factor affecting post-operative recovery of patients [19]. Previously, patients routinely fasted for 3–4 days after gastrectomy, and received gastrointestinal decompression and enteral nutrition support via a nasogastric tube until gastrointestinal function was restored.

In recent years, ELD has been widely applied in clinical practice abroad because of the widespread implementation of ERAS. However, there is a lack of high-level clinical evidence on the safety and reliability of early post-operative oral feeding in GC compared with colorectal cancer [20, 21]. This method has not been used in gastrectomy because of concerns that ELD might directly stimulate the anastomosis site and increase gastric pressure, leading to serious complications such as anastomotic leakage. Recently, an increasing number of centers abroad have started to implement ELD in patients who have undergone gastrectomy and have found it to be safe and feasible [6, 22–24]. However, owing to the lack of high-quality clinical studies in China, we conducted this multicenter study, comprising 11 medical centers, to analyse the effects of ELD and TLD on gastrointestinal function recovery among Chinese patients with GC. Overall, promising clinical results regarding the efficacy and safety of ELD were found, suggesting that ELD could be safely performed in more Chinese centers after gastrectomy.

The first flatus and defecation times after surgery are important clinical indices that reflect the recovery of gastrointestinal function. The results of this study showed that the first flatus time, first defecation time, and post-operative hospital stay in the ELD group were shorter than those in the TLD group, indicating that ELD could promote the recovery of gastrointestinal function in patients with GC after gastrectomy. The reason behind this observation could be that drinking warm water after surgery can stimulate the vagus nerve of the brain and digestive glands, promote the secretion of digestive juice, and accelerate intestinal absorption [25]. Additionally, stimulation of the vagus nerve accelerates contraction of the gastrointestinal smooth muscle, which contributes to better peristalsis of the gastrointestinal tract [26]. Recent studies have observed that early post-operative feeding promotes the recovery of gastrointestinal peristalsis, accelerates gastric emptying, and protects the gastrointestinal mucosa, thereby effectively reducing intestinal bacterial translocation and the risk of intestinal infection [27]. Moreover, ELD can reduce fatigue after laparoscopic DG, improve patients’ medical experience, and lead to faster psychological recovery [28].

ERAS emphasizes early post-operative oral feeding, as opposed to the traditional notion of eating after an anal exhaust. According to a previous consensus, early feeding increases the post-operative intestinal burden and increases the risk of post-operative gastric retention and anastomotic leakage. An important index for evaluating the safety and feasibility of the procedure is the incidence of short-term post-operative complications. In China, there is limited availability of high-quality clinical evidence on the clinical efficacy of ELD after GC surgery, especially in laparoscopic radical total gastrectomy. There was no significant difference in the post-operative surgical complications between the ELD and TLD groups (6.5% vs 9.0%, *P > *0.05). ELD can promote the recovery of gastrointestinal function, and it does not increase the incidence of post-operative complications in patients who undergo either DG or TG/PG. According to conventional views, post-operative fasting and nasogastric tube placement would reduce gastrointestinal pressure and anastomotic edema, providing sufficient time for anastomotic healing. However, such knowledge seems to fade with the publication of the latest evidence. Rossetti *et al*. [29] performed a study involving 145 patients who underwent laparoscopic sleeve gastrectomy; they found that placement of a nasogastric tube did not reduce the incidence of post-operative anastomotic leakage. We hypothesized that the main causes of post-operative anastomotic leakage could be related to uncontrolled diabetes, excessive anastomotic tension, anastomotic ischemia, or anastomotic technical defects. An accurate anastomosis technique would ensure an adequate width of the anastomosis and facilitate accurate mucosa-to-mucosa anastomosis, which is essential to prevent anastomosis leakage. With advances in surgical techniques and perioperative nursing measures, the safety of ELD after gastrointestinal surgery seems to be less concerning.

In addition to anastomotic safety, patient tolerance is also an important consideration in early oral feeding. Slim *et al*. [30] emphasized a significant relationship between feeding intolerance and post-operative complications within 24 h after colorectal surgery. Feeding intolerance appears to be the most predictive of early signs of change after complex surgery. The advantage of this manifestation is evident within 24 h after surgery, which is much earlier than the increase in C-reactive protein or procalcitonin concentration or Dutch leakage score [31]. This was associated with a 5-fold increased risk of complications after the colorectal surgery and a 3-fold increased risk of unplanned reoperation.

Despite the widespread adoption of the ERAS pathway, unplanned readmissions within 30 days after surgery may still occur in some patients. Treatment-related complications, including those resulting from certain treatment measures, such as pulmonary or surgical site infections, may necessitate readmission. Furthermore, patient-related factors, such as age, underlying diseases, obesity, or malnutrition, may increase the risk of readmission. Additionally, poor adherence to post-operative treatment, such as noncompliance with the dietary plan or medication regimen, may lead to readmission. In summary, the causes of unplanned readmissions of ERAS patients within 30 days are varied and require comprehensive assessment and management to ensure optimal patient care.

There are some limitations in our study. First, although PSM was used to balance the baseline characteristics of the two groups of patients, our study was limited by its retrospective nature. More than 60% of the population underwent PG/DG and 70% underwent laparoscopy. Improvements in surgical techniques and perioperative management over time may have influenced the results of this study. With the progress of laparoscopic technology, more people will be treated with laparoscopy, and with the improvement in the detection rate of early GC, the proportion of patients undergoing total gastrectomy may gradually decrease, and the post-operative short-term prognosis of GC will be better. Second, the results of this study suggest that ELD can improve the short-term clinical benefits of post-operative patients with GC; however, further RCTs are needed to be designed to investigate the long-term survival rate and quality of life of patients through follow-up studies. Third, in addition to the stimulation of gastrointestinal peristalsis by ELD, it may also be related to the selection and dosage of anesthetic drugs during operation; therefore, it needs to be verified by further research and design. Furthermore, although many studies have applied ELD to patients after GC surgery and reported improved post-operative outcomes and accelerated recovery, the actual implementation of ELD varies across countries. To date, there is still no consensus on the appropriate timing, composition, frequency, and quantity of ELD, and the feeding plan still needs to be adjusted according to the oral tolerance of patients. Finally, large-scale RCTs are needed to assess the effects of ELD on the regulation of the internal environment of the body.

## Conclusions

The perioperative measures of ELD after radical gastrectomy are not only feasible and safe but also promote the recovery of gastrointestinal function. ELD was associated with shortening flatus time, defecation, and post-operative hospital stay, thereby reducing the hospitalization costs. ELD did not increase post-operative complications, as compared with TLD. Therefore, we suggest that ELD after radical gastrectomy should be used as a standardized perioperative nursing procedure.

## Authors’ Contributions

Y.Z., K.T., J.Y., and J.W. contributed to the conception and design of the research; Y.Z. drafted the manuscript; C.C., Q.Z., S.L., X.Z., L.L., W.W., Q.W., E.L., and Y.Luo performed the research and collected the data; G.Z. participated in the statistical analysis; X.F. and Y.Li reviewed and edited the manuscript. All authors read and approved the final version of the manuscript, and agreed to be accountable for all aspects of the research to ensure that the accuracy or integrity of any part of the work is appropriately investigated.
